# Clinical pattern and outcomes of acute cholangitis among patients at a tertiary health care facility in Kenya: a cross-sectional study

**DOI:** 10.11604/pamj.2026.53.146.52075

**Published:** 2026-03-30

**Authors:** Caleb Karani, Ashley Odiado, Njoroge Maina, Mohamed Onyango, Daniel Ojuka

**Affiliations:** 1 Faculty of Health Sciences, University of Nairobi, Nairobi, Kenya,; 2 Department of Medicine, The Aga Khan Hospital, Kisumu, Kenya

**Keywords:** Cholangitis, biliary obstruction, cross-sectional studies, outcome

## Abstract

**Introduction:**

acute cholangitis (AC) is a life-threatening condition associated with high mortality and lethal complications, with limited data from sub-Saharan Africa. This study determines the clinical pattern and outcomes of acute cholangitis among patients admitted to Kenyatta National Hospital.

**Methods:**

this cross-sectional study retrieved records from the Health Records Department for patients with a discharge diagnosis of AC between 2018 and September 2025. Ethical approval was obtained from the institutional body. We performed consecutive data sampling of all available records. Demographic and clinical data were collected and analyzed using means and proportions. Associations were analyzed using the Chi-square test, and P-values <0.05 were considered statistically significant.

**Results:**

among 111 acute cholangitis patients, the male-to-female ratio was 2:3, and the median age was 61 years. Median length of hospital stay was 12 days, with a 39.6% mortality rate. Malignant obstruction was the predominant etiology (67.6%), followed by benign stricture (11.7%); stent obstruction and stones each accounted for 6.3%. Malignant etiology was associated with worse outcomes (p<0.001), while ERCP drainage was protective (p<0.001). Independent predictors of in-hospital mortality were mental status changes (OR 23.034; 95% CI: 5.356-99.054) and sepsis with multiorgan dysfunction (OR 26.381; 95% CI: 4.028-172.765).

**Conclusion:**

acute cholangitis in our setting is primarily due to malignant biliary obstruction and has high mortality. The strongest predictors of mortality are mental status changes and sepsis. Early diagnosis using Tokyo Guidelines 2018 (TG18) criteria, prompt biliary drainage, and critical care reduce mortality.

## Introduction

Acute cholangitis (AC) is the inflammation of the biliary tree secondary to biliary obstruction. The most common cause of obstruction is choledocholithiasis; other causes include strictures, malignancies, and biliary stent obstruction [1]. Acute cholangitis commonly presents as a triad of fever, jaundice, and right upper quadrant (RUQ) pain, better known as Charcot´s triad, which was first described in 1877 [2]. It can worsen to include mental status changes and hemodynamic instability, after which it is called the Reynolds pentad [3]. Acute cholangitis is diagnosed using clinical characteristics, laboratory results, and imaging findings according to the Tokyo guidelines 2018 (TG18) [4]. Without prompt treatment, it can quickly progress to systemic inflammatory response syndrome (SIRS), septic shock, multiple organ failure, or death. Management depends on disease severity, with the cornerstones being antibiotics and biliary drainage, as stipulated by the TG18 [4]. The incidence of acute cholangitis is between 0.3% and 1.6%, and the proportion with severe cholangitis among these patients is 12.3% [5]. The incidence of cholangitis varies in tandem with the prevalence of cholelithiasis. The global prevalence of gallstone disease is 6%, and in Africa, it is estimated to be between 6.6% and 17% [6,7]. Gallstones are found in 10% to 15% of the white population in the United States. It is much more prevalent in native Americans (60-70%) and Hispanics, but less common in Asians and African Americans. Out of the many patients admitted to the hospital with gallstone disease, 6% to 9% of them are diagnosed with acute cholangitis. Males and females are affected equally. The average age of presentation with AC is 50 to 60 years. In the United States, fewer than 200,000 cases of cholangitis occur per year [1]. The mortality rate has gradually fallen from 100% in the early 1900s to 50% before the 1970s and eventually to 10-30% since 1980, with the introduction of endoscopic biliary drainage [8]. While global mortality rates range between 9.6% and 37%, the specific mortality burden in the Kenyan context remains poorly defined [9-12]. The reported length of stay (LOS) is between 0 and 194 days [13]. There is a scarcity of studies on AC, more so in Kenya and Africa at large. Our specific objectives were to determine the pattern, clinical characteristics, and outcomes of acute cholangitis among patients admitted to Kenyatta National Hospital.

## Methods

**Study design:** a cross-sectional study conducted to determine the clinical pattern, characteristics, and outcomes of acute cholangitis among patients admitted at Kenyatta National Hospital.

**Study setting and population:** the study was conducted at Kenyatta National Hospital (KNH), a public, tertiary, referral hospital in Nairobi, Kenya. With a 2400 bed capacity, it is the largest referral hospital in East and Central Africa. It also serves as a teaching hospital of the University of Nairobi Faculty of Health Sciences and Kenya Medical Training College, among other institutions. The data were collected between 8^th^ September and the 10^th^ of November 2025. This study utilized hospital records between January 2018 and September 2025 of patients above 18 years with a discharge diagnosis of AC according to TG18.

**Variables:** independent variables collected included age, sex, date of admission, date of discharge, and source of referral. Laboratory data collected included the presence of fever > 38°C, white blood cell (WBC) count above or below the reference range, presence of jaundice, elevated total bilirubin, alanine aminotransferase (ALT), aspartate aminotransferase (AST), alkaline phosphatase (ALP), and gamma-glutamyl transferase (γGTP). Data on imaging modality used, presence of biliary dilatation, and the etiology, presence of RUQ pain, vomiting, pruritus, mental status changes, hypotension, and sepsis were also collected. Data on patient management collected included IV antibiotics, fluid resuscitation, and surgical biliary drainage. Lastly, risk factors of mortality, such as age ≥ 65 years and renal dysfunction, were collected. The outcome variable (dependent) was classified into alive upon discharge and in-hospital mortality. Alive upon discharge included patients who were discharged alive and those who were transferred to other facilities, e.g. hospices for further care. We considered possible confounders in our statistical analysis.

**Data source and measurement:** data were extracted from patient records using a Google sheets collection tool, which was pre-tested and refined using an initial sample of 10 files. The tool was developed using the TG18 guidelines [4] and tailored to the study objectives. Baseline laboratory results, such as WBC count above or below the reference range and elevated bilirubin, were obtained from the first tests conducted upon admission. Findings regarding biliary dilatation and the etiology of AC were sourced from imaging reports within the patient files. Clinical signs, including the presence of fever > 38°C and jaundice, were retrieved from triage and admission notes, while remaining demographic and clinical variables were extracted from registration details and clinician history.

**Study size:** as of September 2025, a census of 131 patient files with a primary or secondary discharge diagnosis of acute cholangitis was retrieved from the Health Records department. Of these, 111 files met the study´s inclusion criteria and were included in the final analysis. Exclusions consisted of 18 patient files with insufficient information and 2 pediatric cases (<18 years).

**Statistical analysis:** data analysis was conducted using IBM SPSS Statistics 25. We conducted tests of normality using the Shapiro-Wilk test. We then conducted descriptive analysis and presented baseline characteristics in terms of medians, interquartile ranges, percentages and proportions. The proportional morbidity was estimated by dividing the number of AC cases by the total number of patients admitted to the medical and surgical wards with various conditions within the study period. We used Chi-square and Mann-Whitney U tests for inferential analysis aiming to determine the association between outcome and different variables. We then fed all the significant variables from univariate analysis (p<0.0.5) into a binary regression model to determine risk factors of in-hospital mortality.

**Ethical considerations:** ethical approval was sought from Kenyatta National Hospital-University of Nairobi Ethics and Research Committee and granted prior to commencement of the study (approval number: UP537/05/2025). Good clinical practice and data protection regulations were followed during the conduct of this study. Access to patient files was sought from the Health Information Department. A unique study number was assigned to each patient, and the observations were blinded during analysis.

## Results

**Baseline characteristics:** out of the 111 cases involved in this study, the male-to-female ratio was 2:3, with 44 males (39.6%) and 67 females (60.4%). The median age was 61 years (IQR: 46-71). The proportional morbidity was 0.072%. The median length of hospital stay was 12 days (range 1-197 days). Majority (90.1% of patients) were referred from peripheral facilities (Table 1).

**Table 1:** demographics and clinical characteristics (N=111)

Age in years, median (IQR)	61 yrs (46,71)	Laboratory values	N (%)
**Gender**	**n (%)**	WBC count> ref. range	79 (71.2%)
Male	44 (39.6%)	T-Bil > 21µmol/L	107 (96.4%)
Female	67 (60.4%)	ALT >1.5X ULN	49 (44.1%)
**Referral status**	**n (%)**	AST >1.5X ULN	80 (72.1%)
Yes	100 (90.1%)	ALP >1.5X ULN	96 (86.5%)
No	11 (9.9%)	γGTP >1.5X ULN	98 (88.3%)
**Clinical characteristics**	**N (%)**	**Imaging**	**N (%)**
Fever >38°C	3 (2.7%)	Biliary dilatation	102 (91.9%)
Jaundice	107 (96.4%)	US	49 (44.1%)
RUQ Pain	98 (88.3%)	CT	67 (60.4%)
Vomiting	85 (76.6%)	MRI	20 (18.0%)
Pruritus	75 (67.6%)	ERCP	24 (21.6%)
Mental status changes	38 (34.2%)	MRCP	38 (34.2%)
Hypotension	30 (27.0%)		
Sepsis	40 (36.0%)		
**Management**	**N (%)**		
IV antibiotics	110 (99.1%)		
Fluid resuscitation done	107 (96.4%)		
Surgical biliary drainage	61 (55.0%)		
ERCP management	39 (35.1%)		
PTC	42 (37.8%)		

IQR: interquartile range; RUQ: right upper quadrant; ERCP: endoscopic retrograde cholangiopancreatography; PTC: Percutaneous transhepatic cholangiogram; ULN: upper limit normal; US: ultrasound; CT: computed tomography; MRI: magnetic resonance imaging; MRCP: Magnetic retrograde cholangiopancreatography; IV: intravenous; WBC: white blood cell; T-Bil: total bilirubin; ALT: alanine aminotransferase; AST: aspartate aminotransferase; ALP: alkaline phosphatase; γGTP: Gamma-glutamyl transferase; µmol/L: micromoles per liter

**Figure 1:**
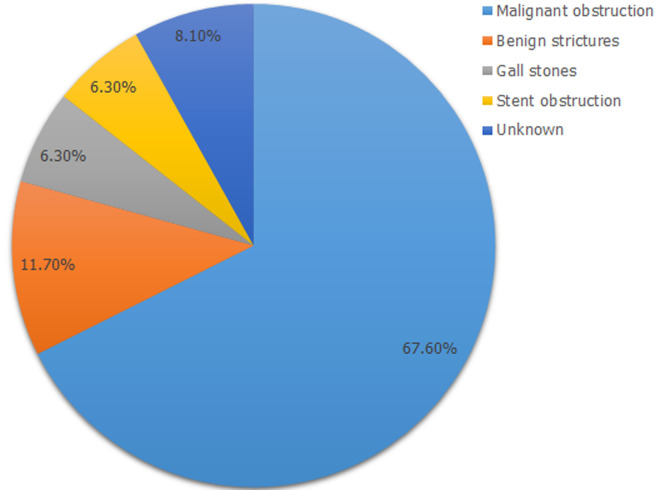
etiology of acute cholangitis

**Clinical characteristics:** a total of 97.3% of the cases were afebrile. White blood cell count was elevated in 71.2% of the patients. Jaundice was present in 97.3%, and RUQ pain in 88.3%. Laboratory investigations revealed elevated cholestatic markers, with high total bilirubin (>21 µmol/L) present in 96.4% (107/111) of the patients. The most frequently elevated liver enzyme was γGTP (>1.5 × URL), in 88.3% of the patients. The least frequently elevated was ALT, observed in 44.1% of cases. Biliary dilatation was present in 91.9% (102 patients). The most frequently utilized imaging modality was abdominal CT, done in 60.4% (67 patients), followed closely by abdominal ultrasound, Magnetic retrograde cholangiopancreatography (MRCP), Endoscopic retrograde cholangiopancreatography (ERCP), and magnetic resonance imaging (MRI). Sepsis occurred in 36% of the patients, hypotension in 27%, and mental status changes were recorded in 34.2% of patients (Table 1). The most common cause of AC was a malignant mass found in 67.6% of the cases (Figure 1). The second most common cause was obstruction due to benign strictures (11.7%), followed by choledocholithiasis and blocked biliary stents each accounting for 6.3% of patients. Eight point one percent (8.1%) of the cases lacked identifiable causes. The in-hospital mortality rate was 39.6% (n=44); notably, 60.4% of patients (n=67) were alive upon discharge.

**Clinical outcomes:** we conducted univariate analysis for the risk factors of in-hospital mortality and included variables such as age, LOS, female gender, age ≥65 years, malignancy as etiology, surgical biliary drainage, mental status changes, renal dysfunction, sepsis with multiorgan dysfunction and hypotension. Malignant etiology was associated with poorer outcomes (49.4% vs 50.6%, p<0.001), while ERCP drainage was protective (82.1% vs 17.9%, p<0.001) (Table 2). We conducted further multivariate analysis by loading all significant variables from the univariate analysis stage into a model. These were mental status changes, sepsis with multiorgan dysfunction, renal dysfunction, malignancy as etiology and hypotension. Out of these, mental status changes and sepsis with multiorgan dysfunction were found to be significantly associated with higher odds of in-hospital mortality (Table 3).

**Table 2:** univariate analysis of risk factors of mortality

Variable	Alive upon discharge (N=67)	In-hospital mortality (N=44)	P-value	Odds ratio (95% CI)
Age, median (IQR)	60 (46 - 71)	63 (56.25 - 72)	0.301	
Length of stay in days, median (IQR)	12 (8 - 26)	7 (4.25 - 22.75)	0.084	
Age ≥ 65 years, n (%)	24 (54.5%)	20 (45.5%)	0.31	1.493 (0.687 - 3.243)
Female gender	41 (61.2%)	26 (38.8%)	0.825	1.091 (0.421 - 1.991)
Malignancy as etiology	39 (49.4%)	40 (50.6%)	<0.001	7.179 (2.304 - 22.38)
Surgical biliary drainage	39 (63.9%)	22 (36.1%)	0.395	0.718 (0.334 - 1.543)
ERCP	32 (82.1%)	7 (17.9%)	0.001	0.207 (0.081 - 0.529)
PTC	21 (50%)	21 (50%)	0.082	2.00 (0.912 - 4.385)
Mental status changes	5 (13.2%)	33 (86.8%)	<0.001	37.2 (11.92 - 116.13)
Renal dysfunction	17 (41.5%)	24 (58.5%)	0.002	3.529 (1.571 - 7.928)
Sepsis with multiorgan dysfunction	4 (11.8%)	30 (88.2%)	<0.001	33.75 (10.23 - 111.30)
Hypotension	3 (10%)	27 (90%)	<0.001	33.88 (9.17 - 125.22)

CI: confidence interval; IQR: interquartile range; ERCP: endoscopic retrograde cholangiopancreatography; PTC: percutaneous transhepatic cholangiogram

**Table 3:** multivariate analysis of risk factors of mortality

Variable	p-value	Adjusted odds ratio (95% CI)
Sepsis with multiorgan dysfunction	<0.001	26.381 (4.028 - 172.765)
Mental status changes	<0.001	23.034 (5.356 - 99.054)
Renal dysfunction	0.739	0.771 (0.167 - 3.552)
Malignancy as etiology	0.083	4.309 (0.824 - 22.524)
Hypotension	0.576	0.550 (0.067 - 4.489)

CI: confidence interval

## Discussion

This study reviewed the pattern, clinical characteristics, and risk factors for mortality of acute cholangitis in our setting. Some of the results were consistent with the global trends based on the studies done on acute cholangitis in the past. Other results differed from global trends, revealing unique trends such as a high mortality rate and the predominance of malignancy as a cause of AC in our setting. Our findings reveal significant clinical patterns that warrant increased vigilance from clinicians managing this life-threatening condition. While fever was uncommon in this study, most patients presented with RUQ pain and jaundice, supported by laboratory findings of cholestasis and imaging evidence of biliary obstruction. Although Charcot´s triad has historically been considered the hallmark of acute cholangitis, the complete triad is frequently absent, particularly in elderly patients and those with severe disease [14-16]. Furthermore, while some studies report fever as the most frequent symptom in over 90% of patients, our study conversely demonstrated that 97% of patients were afebrile on admission [14]. This low prevalence may be attributed to our data collection method which used a single-point-in-time temperature measurement upon arrival. Additionally, this discrepancy likely reflects pre-referral use of antipyretics or antibiotics, either through self-administration or administration at peripheral facilities prior to transfer. Furthermore, the low incidence of fever could be due to the older age of our cohort (median age: 61 years), as advanced age may blunt the febrile response despite severe infection [15,17]. The Tokyo Guidelines 2018 (TG18) recognize the limited sensitivity of clinical features alone by emphasizing the importance of laboratory markers and imaging findings in diagnosis [4,18]. Our findings, therefore, support the applicability of TG18 criteria as a useful diagnostic tool in our setting.

Elevated total bilirubin, ALP, and γGTP indicate advanced biliary obstruction at presentation. Similar patterns have been observed among other cohorts and may reveal delayed presentation or referral from peripheral facilities in our setting [10,13]. A third of the patients in our study displayed advanced symptoms (indicating severe disease), including hypotension, mental status changes, and sepsis with multiorgan dysfunction. Our findings are consistent with previous studies that have been done, demonstrating that severe acute cholangitis is associated with systemic complications and poor outcomes [9,19]. Presence of severe clinical features among patients in our study likely reflects delayed diagnosis and management, referral patterns to a national tertiary hospital, and limited access to early biliary decompression in resource-limited settings. The predominant etiology of acute cholangitis in this study was malignancy-related biliary obstruction, accounting for 67.6% of the cases. In line with our results, a similar study of 801 patients at a German tertiary center reported that 51.9% of episodes were malignancy-related [19]. In contrast, other previous studies consistently identify choledocholithiasis as the leading cause of acute cholangitis [9,13,20,21]. These discrepancies likely reflect differences in study design, eligibility criteria, clinical setting and sample size. The predominance of malignancy in our study likely reflects the role of our facility as a tertiary center managing advanced hepatobiliary disease. This preponderance of malignancy may also be partly explained by the older age distribution of patients in our study (median age: 61 years), as the incidence of hepatobiliary malignancies increases with advancing age [22-24].

Due to limited cancer screening in low- and middle-income Countries, malignancy-related cholangitis may reflect late-stage presentation of hepatobiliary cancers. These often cause prolonged, silent biliary obstructions and are diagnosed after infection-related complications have developed. Malignant etiology was associated with poorer outcomes compared to stones, stent obstruction and others which is consistent with previous studies [18,19,25,26]. In addition, sepsis with multiorgan dysfunction, hypotension, mental status changes and renal dysfunction were the strongest independent predictors of in-hospital mortality. These are manifestations of multiple organ failure likely caused by sepsis [8]. Consistent with our findings, Schneider et al. also linked hypotension to mortality, noting that mental status changes represented the strongest predictor of mortality [19]. Kimura *et al* noted that multiple organ failure with irreversible shock is the leading cause of death in patients with AC [8]. With the advent of surgical biliary decompression in 1903, the mortality rate was almost 100%. It remained at 50% before the 1970s and fell to 10%-30% since 1980, attributable to the introduction of endoscopic biliary decompression techniques [8]. The current mortality rate ranges between 9.6% and 37% [9-12]. The mortality rate of 39.6% is above this range. This is likely because the most common cause of AC in our study was malignancy-related as compared to previous studies where majority of patients had gallstones [9,11,13,21]. We also postulate that late presentation to our facility, delayed and sometimes lack of endoscopic biliary decompression and financial limitations which hinder patients from receiving timely healthcare services could have played a part. Patients who underwent ERCP management had a lower mortality rate compared to those who did not. Timely ERCP has been proven to be a cornerstone in the management of acute cholangitis [4,27,28]. Our findings align with previous studies which generally link ERCP with improved outcomes and supports TG18 recommendation for prompt biliary drainage [28-30]. This study has limitations borne of its cross-sectional design and the sample size. It relies solely on existing patient data and it cannot establish causality. Additionally, it was a single center study therefore reducing its generalizability. To our knowledge, this is the first study on AC in our setting, thereby providing a unique perspective to the existing literature on the presentation and challenges associated with AC.

## Conclusion

Acute cholangitis, most commonly caused by malignant biliary obstruction, is associated with a high mortality rate. Many patients do not present with the classic Charcot´s triad, and therefore TG18 is more useful in diagnosis. Mental status changes and sepsis with multiorgan dysfunction were the strongest independent predictors of in-hospital mortality highlighting the importance of early recognition of severe disease. Timely access to biliary drainage, such as ERCP management, and critical care may decrease mortality from acute cholangitis in this setting.

### What is known about this topic

- Acute cholangitis (AC) is an inflammation of the biliary tree secondary to obstruction from stones, malignancy, or other etiologies, with the Tokyo Guidelines 2018 (TG18) widely used for diagnosis;
- It carries a high mortality rate most commonly due to refractory septic shock;
- There is a paucity of data from sub-Saharan Africa.

### What this study adds

- This study reports a mortality rate higher than what the current literature reports and identifies malignancy as the leading cause of AC;
- Multivariate analysis identified mental status changes and sepsis with multiorgan dysfunction as the strongest predictors of in-hospital mortality.
